# Pain management with epidural catheter and epidural analgesia after spinal dorsal instrumentation of lumbar spine

**DOI:** 10.1097/MD.0000000000032902

**Published:** 2023-02-17

**Authors:** Motaz Hamed, Harun Asoglu, Tim Lampmann, Lena Marie Winkelmann, Abdallah Salemdawod, Martin Müller, Hartmut Vatter, Mohammed Banat, Lars Eichhorn

**Affiliations:** a Department of Neurosurgery, University Hospital Bonn, Bonn, Germany; b Department of Anesthesiology and Intensive Care Medicine, University Hospital Bonn, Bonn, Germany; c Center for Advanced Imaging Research, Department of Diagnostic Radiology and Nuclear Medicine, University of Maryland Marlene and Stewart Greenebaum, Comprehensive Cancer Center, University of Maryland, Baltimore, MD; d Department of Emergency Medicine, Inselspital, Bern University Hospital, Bern University, Bern, Switzerland; e Clinic for Anesthesiology and Intensive Care Medicine, Helios Klinikum Bonn/Rhein-Sieg, Bonn, Germany.

**Keywords:** early postoperative complications, epidural analgesia, intravenous systemic analgesia, postoperative pain, spinal dorsal instrumentation

## Abstract

Spinal dorsal instrumentation (SDI) is an established treatment for degenerative spinal diseases. Adequate and immediate postoperative pain control is important for patient recovery and may be compromised by uncertainty about its efficacy and concern about early postoperative surgical complications or adverse events. The aim of the current study was to compare the use of epidural analgesia (EA) with systemic analgesia (SA) as regards pain reduction and early postoperative complications after SDI. Pain management with epidural or systemic analgesia in patients undergoing SDI by posterior approach between January 2019 and July 2020 was evaluated by clinical functional testing, measuring total opioid amounts used, and evaluating numerical rating scale values 24 and 96 hours postoperatively. The following were also monitored: demographic data, number of affected segments, length of hospital stay, inflammatory markers (leukocytes and serum C-reactive protein), early postoperative surgical complication rates, and adverse events. In total 79 patients were included (33 in the EA and 46 in the SA group). The SA group had significantly lower numerical rating scale values at days 1 to 4 after surgery (*P* ≤ .001) and lower cumulative opioid use than the EA group (*P* < .001). We found no difference in infection parameters, length of hospital stay or surgery-related complication rates. Our data demonstrate that epidural anesthesia was inferior to an opioid-based SA regime in reducing postoperative pain in patients undergoing spinal surgery. There is no benefit to the use of epidural catheters.

## 1. Introduction

Spinal dorsal instrumentation (SDI) is an established treatment for degenerative spinal lumbar diseases in adults.^[1–3]^ Postoperative pain is one of the most common concerns of patients after spine fusion surgery with instrumentation.^[4,5]^ Effective postoperative pain management affects quality of life and early recovery, and also influences the extent of postoperative morbidities and the length of hospital stay.^[6–8]^ Additionally, prolonged postoperative pain stimulates the autonomous nervous system, which reacts by releasing inflammatory cytokines and stress hormones like epinephrine, corticotrophin, and systemic cortisol.^[9,10]^

Some patients who suffer from chronic pain after SDI may misuse opioid analgesics and tranquilizer medication, which may lead to high tolerance and/or addiction.^[11,12]^ Postoperative pain management is essential to improve patients early mobility after surgical treatment and to reduce the risk of complications; therefore multiple analgesic strategies are used, but no consensus guidance has been established.^[6,13,14]^

Epidural analgesia (EA) and intravenous (IV) systemic analgesia (SA) have spread in clinical practice as postoperative pain treatment and proved to be sufficient.^[10,11,15]^ One systematic review showed the analgesic benefits of EA in spinal surgery,^[13]^ whereas other authors only found modest and inconsistent differences between the 2 procedures.^[16–18]^

This study aims to compare EA and SA after SDI to clarify which method was better and more effective for pain control and pain reduction at our spine center. We also considered adverse effects and surgery-related postoperative complications.

## 2. Methods

### 2.1. Patient selection and inclusion criteria

**Figure 1.**
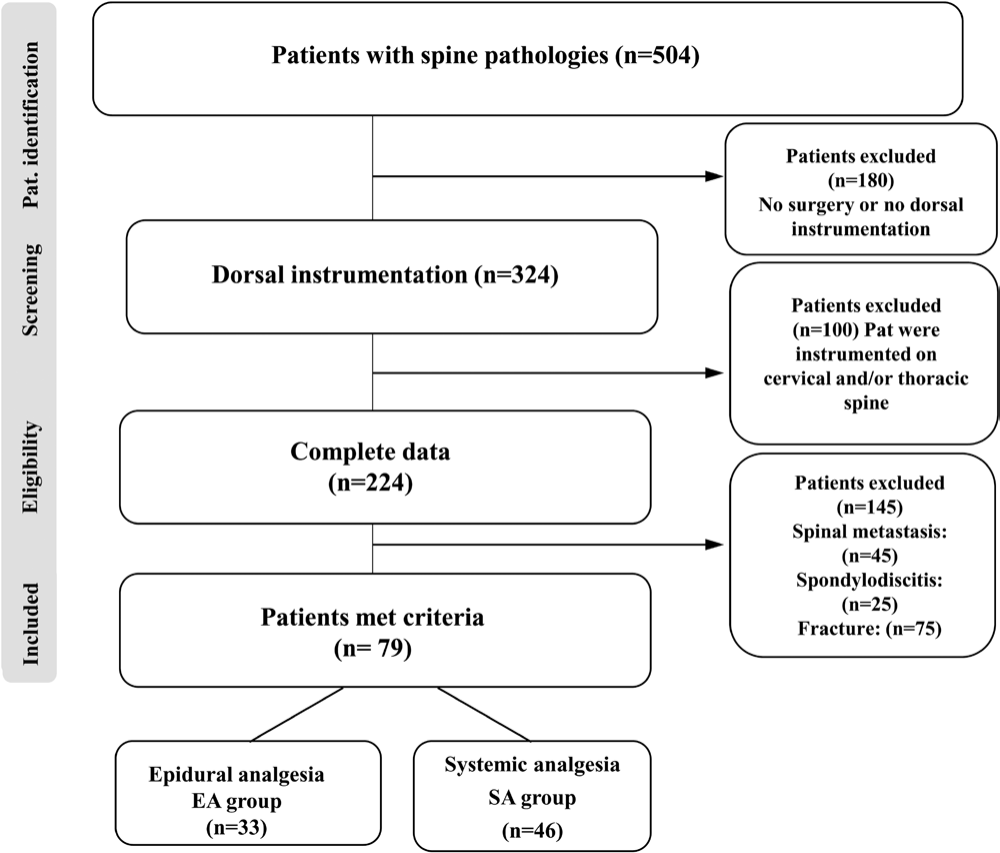
Flow chart showing our algorithm and inclusions criteria.

This retrospective single center cohort study evaluated postoperative pain management and analgesia regimes (i.e., epidural–EA- vs intravenous analgesia–SA-) in patients at our spine center after spinal posterior open instrumentation with or without interbody fusion of the lumbar spine between January 2019 and July 2020. Indications and inclusion criteria for surgery were spinal canal stenosis with spondylolisthesis and degenerative disc disease, all patients should have symptoms as back and/or leg pain, they should be treated maximal conservative with medication and physiotherapy before the operation, and the patients have no quality of life because of pain or neurological symptoms. Exclusion criteria were an incomplete medication history, an anesthetic or opioid allergy, and a history of chemical dependency. Furthermore, patients with other spinal pathologies (infection, tumor, fracture) were excluded. Figure 1 shows our algorithm and inclusions criteria.

Records of patients undergoing spinal surgery were screened for the following: demographic data (age, sex, and body mass index [BMI]), type of analgesia regime, number of operated levels, operation duration, numerical rating scale (NRS) score, amount of opioid use, postoperative time between surgery and mobilization, preoperative use of opioids, and total length of hospital stay. Preoperative systemic inflammatory laboratory parameters such as white blood cell count and serum C-reactive protein were documented. Standard laboratory investigation of inflammatory markers (as previously described^[19]^ was undertaken in our center. Early postoperative complications (both surgery-related and adverse events) were assessed using a publicly available list from the Agency for Healthcare Research and Quality and the Center for Medicare and Medicaid Services. They are referred to as patient safety indicators and hospital-acquired conditions.^[20–22]^

### 2.2. Periprocedural analgesia concepts and pain management

All patients were treated prior to surgery with oral pain medication according to world health organization guidelines.^[23]^ Postoperatively each patient received as standard a total of 1600 mg ibuprofen (3 × 600mg), 1500 mg paracetamol or 90 mg codeine (3 × 500 or 3 × 30mg), 750 mg methocarbamol in the evening for muscle relaxation, and 40 mg pantoprazole, regardless of which analgesia regime was used.

In the case of patients receiving epidural analgesics, a 16-gauge Portex catheter was placed in the epidural space under direct vision on completion of the surgical procedure in the operation segments or 1 segment above it. The epidural catheter was threaded through a skin puncture about 3 cm away from the surgical incisions to avoid inadequate surgical wound compression. Ropivacaine (2 mg mL^-1^) was administered via an automatic pump with a starting rate of 14 mg/hour. Postoperative pain was measured with the NRS and a cold discrimination dermatomal test was performed 3 times per shift for the first 24 hours, then once per shift for the next 72 hours. Where pain relief was inadequate, the infusion rate was increased until functional failures occurred such as motor disorder or paresthesia. Patients could additionally receive parenteral piritramide (7.5 mg piritramide and 100 mL 0.9% saline solution) via a 15 to 30 minutes intravenous infusion in the interval of 4 to 6 hours. The epidural catheter was removed after the third day in all cases. Before removing the EC, Ropivacaine was reduced in steps of 2 to 4 mg according to the patient wishes.

In the group with SA, all patients received piritramide on demand (7.5 mg piritramide and 100 mL 0.9% saline solution, 15 to 30 minutes intravenous infusion).

To avoid respiratory failure, a mandatory [blood oxygen saturation (SpO_2_)] minimum 45 minutes interval was. routinely measured in both groups.

### 2.3. Postoperative pain evaluation and documentation

The NRS was used to measure pain during the study, additionally patient-specific functional scale.^[24,25]^ Back pain functional score were evaluated Pre-Surgery and 3 months after surgery.^[26]^ Observations were recorded every 8 hours. For this study a median NRS score was calculated each day to measure the efficacy of analgesia. To eliminate as much as possible bias due to the skill or experience of the surgeon, operations were carried out by only 4 neurosurgeons in the center; 2 of them treated patients with epidural analgesia and the other 2 prefer the postoperative therapy with systemic analgesia. The NRS scores in both groups were analyzed retrospectively. Confounders were: sex, age at surgery, BMI, duration of surgery, surgery on > 3 motion segments, pretreatment with opioids, pretreatment with non-steroidal anti-inflammatory drugs, postoperative complications, and need of revision. We chose not to include data on the patients feelings about their treatment preferences.

All procedures performed were in accordance with the ethical standards of the institutional and national research committee (Ethic committee of the Rheinische Friedrich Wilhelm University Bonn) and with the 1964 Helsinki declaration and its later amendments or comparable ethical standards. Study design: Retrospective clinical cohort study. The investigation was approved by the local ethics committee (protocol no. 084/21).

### 2.4. Statistical analysis

All analyzed data were performed using IBM^®^ SPSS^®^ Statistics V22.0 (IBM, Chicago, Illinois). In the descriptive analysis, the median distribution of continuous variables is presented along with the interquartile range (interquartile range, 25^th^–75^th^ percentile), and the total number of categorical variables per category is presented along with the percentage. In the case of categorical variables, data are given as numbers and percentages. After normality testing via the Shapiro–Wilk test, continuous normally distributed data were compared using *t* tests, while the Mann–Whitney *U* test was used for non-parametric data. Nominal data was tested between groups using the Fisher´s exact test and in case of multinomial data with a Chi^2^-test. Univariate analysis using Fisher exact (2-sided) and independent *t* test was conducted. A *P* < .05 was considered significant.

## 3. Results

A total of 79 patients were surgically treated between January 2019 and July 2020 and included in the study. The patients, who were aged 47 to 87 years, underwent reconstructive thoracolumbar surgery. Table 1 shows the baseline patient data in the upper part.

**Table 1:** Univariable analysis of patient characteristics and surgical procedures [using Fisher exact (2-sided) and independent *t* test].

Total (N = 79)		EA group	SA group	*P* value
No. of patients		33 (42%)	46 (58%)	
Age (yr), median [q1–q3]		75.0 [65.0–79.0]	72.5 [61.0–77.0]	.438
Gender Female Male		17 (51.5%) 16 (48.5%)	21 (45.7%) 25 (54.3%)	.607
BMI, kg/m^2^, median [q1–q3]		25 [22–29]	29 [26–35]	.003*
Length of stay in days, median [q1–q3]		14 [10.0–19.0]	13 [9.0–24.0]	.913
Duration of operation in min		337.0 [280.0–432.0]	310.0 [243.0–375.0]	.183
Full mobilization				<.001*
	Within the first 24 h of operation	10 (30.3%)	43 (93.5%)	
	24–48 h after surgery	23 (69.7%)	3 (6.5%)	
Surgery-related complications				.073
	Temporary. neurological deficit	2 (6.0%)	1 (2.1%)	
	Cerebrospinal fistula	2 (6.0%)	1 (2.1%)	
	Wound infection	3 (9.0%)	1 (2.1%)	
	Disturbance of wound healing	2 (6.0%)	2 (4.3%)	
	Postoperative bleeding	1 (3.0%)	2 (4.3%)	
In-hospital complications				.834
	Pneumonia	2 (6.0%)	1 (2.1%)	
	UTI	1 (3.0%)	2 (4.3%)	
	Mortality	1 (3.0%)	1 (2.1%)	.998
Revision		4 (12.1%)	4 (8.69%)	.619
Average ropivacaine per h		10 [10.0–10.0]	0 [0.0–0.0]	<.001*
No. patients needing additional postoperative opioids		30 (90.9%)	35 (76.1%)	<.089
Stabilized level				.834
	1 Level	6 (18.2%)	4 (8.7%)	
	2 Levels	8 (24.2%)	7 (15.3%)	
	3 levels and more	19 (57.6%)	35 (76.0%)	
NRS, median [q1–q3]				
	Pre surgery	8 [7–10]	8 [7–10]	1.0
	Directly after surgery	6 [5–7]	6 [5–7]	1.0
	First day after surgery	6 [5–7]	4 [4–5]	<.001*
	4th day after surgery	3 [3–4]	2 [2–3]	.004*
PSFS, median [q1–q3]				
	Pre surgery	7 [7–9]	8 [7–10]	.999
	D4 after surgery	5 [3–7]	4 [2–6]	.781
	At discharging	3 [3–6]	2 [2–4]	<.003*
Systemic opioid consumption after surgery (in mg)				
	D1	30	15	<.001*
	D2	45	22.5	.002*
	D3	45	22.5	<.001*
Systemic inflammatory laboratory parameters				
	CRP (pre surgery), mg/dL	3.3 [1.0–5.7]	4.2 [0.9–9.4]	.142
	CRP (D3), mg/dL	27.8 [12.8–53.1]	30.6 [10.6–67.6]	.657
	WBC (pre surgery), m/mm³	6.9 [5.7–8.0]	7.1 [6.3–8.2]	.830
	WBC (D3), m/mm³	8.7 [7.5–12.8]	8.0 [6.2–9.3]	.810
BPFS, median [q1–q3]				
	Pre surgery	30 [22–44]	32 [21–45]	.799
	3 mo after surgery (30 patients of EA group, and 40 Patients of SA group)	44 [37–55]	45 [33–57]	.852

Categorical variables are shown as number (%) and continuous variables as median [interquartile ranges].

AEs = adverse events, BMI = body mass index, BPFS = back pain functional score, CRP = C-reactive protein, D = day, EA = epidural analgesia, min = minutes, NRS = numerical rating scale, PSFS = patient-specific functional score, q1–q3 = first quartile–third quartile, SA = intravenous systemic analgesia, UTI = urinary tract infection, WBC = white blood cell count.

* *P* ≤ .05: statistically significant.

**Figure 2.**
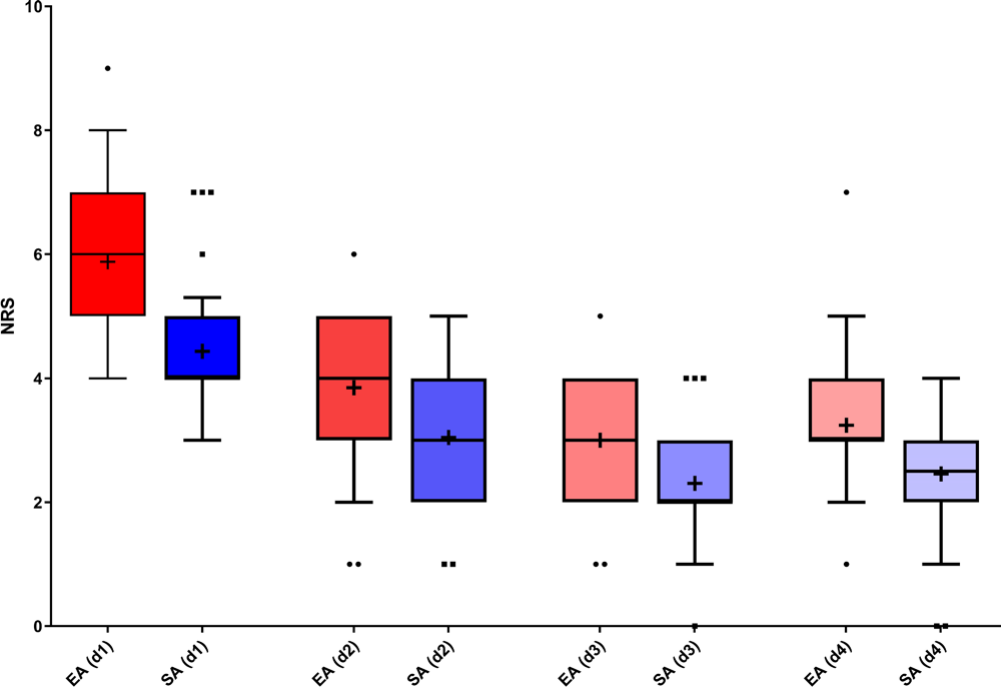
(Box and whiskers): Numerical rating scale (NRS) for postoperative pain between day 1 and day 4 after surgery in both groups. The 25^th^ and 75^th^ percentile of the data defines the box portion. The line inside the box is the median (the 50^th^ percentile). The mean is as (+) identified and the whiskers are defined by 10^th^ and 90^th^ percentile. NRS = numerical rating scale.

Our patients were divided into 2 groups, with the EA group receiving epidural analgesia via epidural catheter, and the SA group receiving IV systemic analgesia. Table 1 shows additionally the univariable analysis of characteristics and surgical procedures between the 2 groups. The percentage of patients who needed additional opioids after surgery was in total 82%. Against our expectations we found significant differences between the 2 groups regarding opioid consumption: patients with EA needed more IV analgesia (*P* ≤ .001) and felt more pain from the first day after the operation (*P* ≤ .001) until the 4th day (*P* = .004). The percentage of patients who needed additional opioids after surgery was 90.9% in the EA group and 76.1% in the SA group. Figure 2 shows the difference in numerical rating scale (NRS) scores for pain between day 1 and day 4 after surgery. In addition to the NRS, the patient-specific functional scale score was collected to assess activity in addition to pain after surgery and at discharge. There was also a difference between the 2 groups, especially on the day of discharge. Patients with SA had a significantly higher BMI (*P* = .003), were able to leave their beds in < 24 hours after surgery compared to 24 to 48 hours with epidural analgesia (*P* ≤ .001), and needed less opioids (i.e., piritramide) in total (*P* ≥ .001). We found no difference in blood cell count or C-reactive protein after surgery between the groups. Furthermore, we found no significant difference between the groups regarding hospital length of the stay or surgery-related parameters such as the number of stabilized segments, duration of operation, or complications; there was also no difference in the rate of adverse events, the revision rate or mortality.

Furthermore, we found no significant difference between the 2 groups in the short-term 3-month follow-up after surgery. There is an overall improvement in function (back pain functional score pre surgery EA/SA 30/32, and 44/45).

## 4. Discussion

Multilevel lumbar spine surgery results in severe pain lasting into the second postoperative day, with moderate pain persisting for up to 1 week after surgical treatment.^[6,11,27]^ Since the 1980s, many studies involving adult patients have compared the effectiveness of epidural and IV systemic analgesia, although not specifically considering patients with spinal surgery.^[28–31]^ The optimal pain management and analgesia treatment after lumbar spine surgery with instrumentation and fusion in combination with spinal canal decompression remains a subject of debate.^[32,33]^ Intrathecal analgesia approaches are various and include long-acting amide local anesthetics, intrathecal morphine, or a combination of both.^[34]^ Systemic analgesia uses a combination of non-steroidal anti-inflammatory drugs and stronger opioids.^[35]^ In the more recent past analgesic adjuncts such as ketamine, gabapentin or dexmedetomidine have shown benefit.^[15]^ While some reviews recommend epidural analgesia in spinal surgery,^[36–38]^ some authors found only a modest and inconsistent clinical effect in pain reduction using epidural compared to intravenous SA techniques.^[17,37]^

In this study, the epidural analgesic solutions revealed no significant improvement in postoperative pain control over IV analgesics alone for patients with posterior spinal fusion. We found a significant higher NRS score in the first 4 days for patients with EA than with SA, suggesting that the epidural option provided inadequate pain relief in patients after spinal surgery. This finding is contrary to our hypothesis, that patients with EC and supplementary SA would need less opioid medication than patients with SA alone. Murphy et al^[39]^ described the use of epidural buprenorphine versus intramuscular morphine after spinal fusion surgery and reported that both produced excellent, equivalent analgesia.

The efficacy of an epidural analgesic should be indicated by the amount of IV medication consumed. In our study, patients with EA required supplemental IV piritramide to provide adequate analgesia after posterior spinal fusion surgery. Although there is a debate about whether EA provides a benefit over SA,^[38,40]^ our cohort showed the opposite. On the 1 hand, the patients may not receive enough pain medication from the epidural catheter, although 2 of 33 patients already suffered from temporary neurological deficit. It tempting to speculate, that the dorsal surgical approach might affect the posterior branches of the spinal nerves and that nerval trauma cause pain itself. Patient might more likely benefit from sedative and anxiolytic effects of the opioids itself then from the analgetic effects. Our hypothesis for the delayed mobilization of the EA group is the patients fear of dislocating the catheter. The fear of mobilization combined with the high need for pain medication resulted in the high dose of opioids and high NRS scores seen in our data on the group with the epidural catheter. Another possible reason why patients in the EA group needed more IV piritramide was that, this group of patients was more controlled due to the EC and were asked more often for pain and analgesia. We also think that the placing and localization of EC also played a major role in increasing the need of additionally IV analgesia in the EA group.

Although intrathecal opioids have numerous advantages over systemically used opioids, reported side effects are similar to systemic opioid analgesia and include urinary retention, pruritus, nausea, unreliable delivery systems, and respiratory depression.^[41]^ A side effect of epidural local anesthetics used in patients undergoing posterior spinal fusion surgery is thigh numbness.^[39]^ Purnell^[42]^ reported a case in 1982 in which a patient received epidural bupivacaine 0.25% (divided doses for a total of 60 mL over approximately 6–7 hours) that resulted in motor block lasting for 9.5 hours.

In a small prospectively randomized comparison Cassady et al^[43]^ evaluated thoracic EA versus IV analgesia in adolescents undergoing posterior spinal fusion. Although both methods were comparably effective and safe, the return of bowel function with EA accelerated recovery and speed of hospital discharge. They left this finding open to interpretation, but felt it could suggest that a primary limiting factor in postoperative recovery (i.e., gastrointestinal dysfunction) is favorably impacted by epidural analgesia.

The results of our data have changed our procedure. We do not use more EC after dorsal instrumentation. We are in the process of establishment an algorithm for SA, we hope to publish these results in the future.

## 5. Limitations

The present study has several limitations. Acquisition of data was retrospective. Furthermore, patients were not randomized, but treated according to the expert opinion of their neurosurgeon. Additionally, the present data represent only a single center experience. We have placed the catheter on the operated segments, or just 1 segment above it, this may have been the major reason why the patients who received epidural analgesics had inferior outcomes in this study. Other main limitation is the small number of cases in each group. Thus, the study might not have had sufficient power to detect smaller differences between the groups.

## 6. Conclusions

Our Data demonstrate that our cohort has no addiotinally therapeutic benefit from EA with EC. Based on our results, the implantation of the EC schould be discussed critically and open with the patients. Sufficient postoperative SA seems usually to perform well on its own. Our recommendation meets the criteria for a level of evidence 3, based on the publication by Kaiser at al.^[44,45]^

## Author contributions

**Conceptualization:** Motaz Hamed, Mohammed Banat, Lars Eichhorn.

**Data curation:** Motaz Hamed, Lars Eichhorn.

**Formal analysis:** Tim Lampmann, Martin Müller, Lars Eichhorn.

**Methodology:** Motaz Hamed.

**Project administration:** Motaz Hamed, Mohammed Banat.

**Resources:** Motaz Hamed, Hartmut Vatter.

**Supervision:** Mohammed Banat, Lars Eichhorn.

**Validation:** Mohammed Banat.

**visualization:** Mohammed Banat.

**Writing – original draft:** Lars Eichhorn.

**Writing – review & editing:** Motaz Hamed, Harun Asoglu, Tim Lampmann, Lena Marie Winkelmann, Abdallah Salemdawod, Mohammed Banat, Lars Eichhorn.
